# A Model for Geometry-Dependent Errors in Length Artifacts

**DOI:** 10.6028/jres.117.013

**Published:** 2012-09-24

**Authors:** Daniel Sawyer, Brian Parry, Steven Phillips, Chris Blackburn, Bala Muralikrishnan

**Affiliations:** 1 National Institute of Standards and Technology, Gaithersburg, MD 20899; 2 The Boeing Company Seattle, WA 98108

**Keywords:** ASME B89.1.12, ball bars, Coordinate Measuring Machine (CMM), design of length artifacts, dimensional artifacts, ISO 10360, high accuracy length standards, performance evaluation of (CMM), Step Gauges

## Abstract

We present a detailed model of dimensional changes in long length artifacts, such as step gauges and ball bars, due to bending under gravity. The comprehensive model is based on evaluation of the gauge points relative to the neutral bending surface. It yields the errors observed when the gauge points are located off the neutral bending surface of a bar or rod but also reveals the significant error associated with out-of-straightness of a bar or rod even if the gauge points are located in the neutral bending surface. For example, one experimental result shows a length change of greater than 1.5 µm on a 1 m ball bar with an out-of-straightness of 0.4 mm. This and other results are in agreement with the model presented in this paper.

## 1. Introduction

This paper discusses an error source associated with the bending of long length artifacts due to gravity. This error is distinguished from other effects such as “beam sag,” which is a second order error [1], and from artifact deflection under probing forces, which can convert second order errors into first order errors [2]. Specifically, it is a generalized form of the errors associated with artifact deflection, which includes the error associated with placing gauging points off the neutral axis of the artifact as a special case [2]. More interestingly, it is shown that even for artifacts where the gauge points intersect the neutral bending axis, e.g., the center of a round rod used for a ball bar, nonlocal geometrical effects, such as poor straightness, can still produce a significant length error. The magnitude of this error depends on the nature of the out-of-straightness of the artifact. Specifically, large out-of-straightness leads to larger errors.

Fortunately, with knowledge about this error source, it is easy to construct long artifacts that make this error negligible. However, ignoring this principle may result in producing a long (greater than 700 mm) artifact that may have either a slight bend, or spheres not well centered on the end of a ball bar, or some combination of these geometric errors. Specifically, an artifact bar that has sub-millimeter straightness or centering errors may change its length by a micrometer when physically rotated about the long axis. Using such a ball bar to evaluate the measurement performance of a Coordinate Measuring Machine (CMM) may result in erroneous CMM performance evaluations. Although deformation of straight and uniform artifacts has been described for end standards such as gauge blocks, e.g., the use of Airy and Bessel points [3–4], their applicability to artifacts of general geometry has not been sufficiently addressed in the literature. Moreover, the sensitivity of artifact length variations due to curvature has not been carefully studied or described. This paper provides a detailed description of this behavior and a mathematical model of this source of long-artifact length variation. Physical measurements of this behavior and a comparison to the mathematical model predictions are also provided. This paper is motivated by the observation that several 1 m long ball bars recently examined at NIST by NIST personnel all were found to have at least one of the aforementioned geometric errors.

## 2. Background

**Fig. 1:**
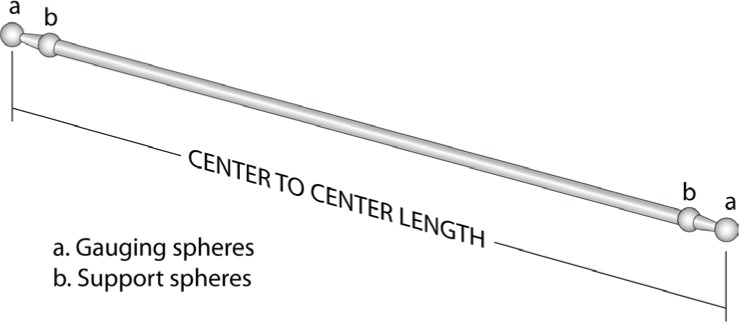
Schematic of ball bar.

Ball bars and step gauges are dimensional artifacts that are used primarily for performance characterization of Coordinate Measuring Machines (CMMs) [5–7]. These artifacts consist of a rigid support structure with precisely defined gauge points. Step gauges typically have these gauge points located in the center of small flat discs. For ball bars, they are the centers of the spheres attached at each end of the support bar (Fig. 1). The critical feature of interest is the straight-line distance between the two gauge points, e.g., the sphere-center-to-sphere-center length for ball bars.

**Fig. 2:**
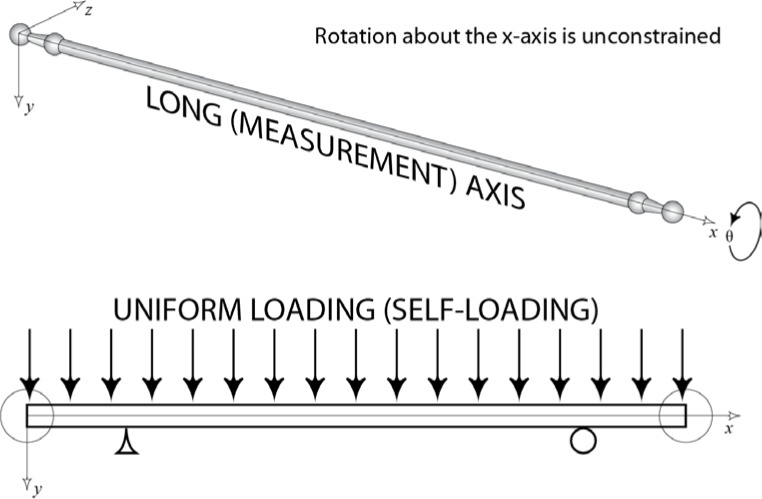
Simplified model of a ball bar as simply-supported beam with a circular cross-section and uniform loading (self-loading).

The use of long artifacts in CMM volumetric performance tests requires measuring the artifact in various horizontal, vertical and inclined positions. The specific rotational orientation of the artifact about its long or measurement axis (*x*-axis in Fig. 2) is not typically specified. Because of imperfect construction of these artifacts, it may be necessary to specify this orientation in order to assure that the length of the artifact is consistent during performance tests. For example, during the calibration of a 1 m ball bar at NIST, it was documented that the ball bar changed in length by more than 1.5 µm when the bar was rotated 180° about its measurement axis (this corresponds to rotation *θ* about the *x*-axis in Fig. 2). Similar behavior was documented while performing measurements using several different bars.

These errors are a result of the out-of-straightness condition of the artifact. For a specified orientation (both rotational and with respect to gravity) the length of the artifact is well defined and can be calibrated with a small uncertainty. However, if its subsequent use is in a different orientation, then changes in the artifact’s length may occur. Many ball bars are produced that are nominally symmetric about their measurement axis, e.g., circular cross sections, consequently subsequent use of these artifacts is typically in a random rotational orientation. Similarly, rectangular cross sectional artifacts are sometimes placed on their side (rotated 90 degrees) to allow CMM probing with an offset probe stylus. Unless these artifacts have been manufactured to be straight, the orientation effect described in this paper may cause significant errors.

This paper primarily discusses the case of a circular cross section ball bar mounted in the horizontal position; however the error can occur for any cross sectional geometry. The largest range of length variation happens when an artifact is mounted in the horizontal orientation, so analysis of other positions will be of smaller magnitude. If a more detailed understanding of the length variation is desired, it is easy to extend the method developed in this paper to the more general case.

**Fig. 3:**
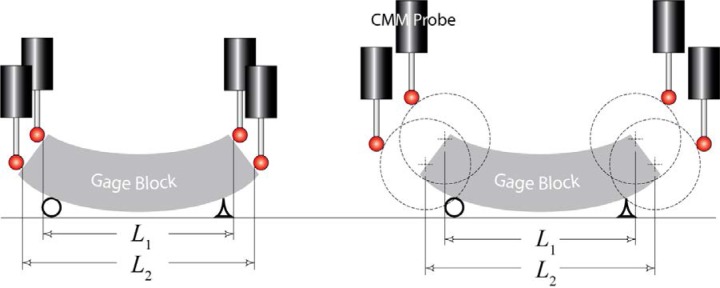
The illustration on the left shows the inspection setup of a simply supported gauge block using corresponding points, one on each face. The illustration on the right shows an analogous inspection setup using a ball bar with the sphere centers located at the position of the gauge points described on the left hand side of the figure. The curvature of the bar and the slope of the end-faces are a result of gravitational forces and not the geometry of the bar. In each case, *L*_1_ < *L*_2_. These effects are exaggerated for illustrative purposes.

One aspect of the length variation in artifacts is well known and can be described using a simple model of a gauge block. These blocks are one of the most commonly used standards in dimensional metrology. They are simple prismatic artifacts of known length with parallel end faces. The calibrated length of a gauge block is defined as the distance between two gauge points that are located on opposite end faces. (This definition is not exactly consistent with the definition of length of gauge blocks described in the American Society of Mechanical Engineers B89.1.9-2002 Gauge Blocks documentary standard but for our illustrative purposes we will use this simplified definition of the length of a gauge block.) Small deviations in the slope of one end face relative to the other along with slight inaccuracies in the probe location may lead to an apparent length measurement error; it is important that the end faces be sufficiently parallel to make this error source negligible. Figure 3 shows the orientation of the end faces, due to gravity, of a simply supported gauge block. (These blocks are nominally straight. The exaggerated curvature and slope of the end faces in the figure are a result of gravitational forces and not the geometry of the bar.) A length in this example is defined as the distance between two points on opposite end faces of the gauge block that are at the same relative position on each end face. Given this definition, the illustration on the left side of Fig. 3 shows the measurement of two individual lengths: *L*_1_ and *L*_2_. These lengths are not equal because of the relative slope of the end faces.

Maintaining parallelism between the two end faces of gauge blocks requires particular mounting conditions. Specifically, the magnitude and direction of the slope of the end face is dependent on the relative position of the gauge block support points. The so called Airy Points [3] specify the location of the mounting points which produce slopes of the end faces that are equal and vertical. In practice, gauge blocks are supported on the Airy Points when inspected using a CMM. However, ball bars are typically supported near the gauging spheres for purposes described in Phillips [2].

For ball bars, this source of length variation is analogous to the errors in the measurement of gauge blocks as described above. This point should be clear if one simply understands that the sphere center of the gauging spheres on a ball bar and the gauge point on a gauge block perform identical functions, i.e., act as reference points for length measurements. This can be seen in the illustration on the right side of Fig. 3. The spheres in this illustration are positioned so that the sphere centers correspond to the gauge points depicted on the left side of the figure. The primary difference between inspecting a gauge block as opposed to a ball bar is that the sphere center is fixed relative to the end of a ball bar. So if the spheres are not positioned precisely along the centerline or centroid of the cross-section of the ball bar (analogous to not probing the gauge block exactly along the centerline), the inspected length will vary. This is because, when viewing the bar along a horizontal plane, the sphere position relative to the centerline of the bar is dependent on the angular orientation of the bar about the measurement axis during measurement. This angular orientation is often arbitrarily selected when positioning a ball bar during measurements.

## 3. Model

The preceding section described one very simple source of possible length variations in straight ball bars. This section will focus on deriving a general mathematical model that describes the length variation of ball bars, which is not limited to perfectly straight ball bars with the sphere offset from the center of the end face. In particular, even if the gauge point (e.g., sphere center) is mounted directly in the center of the bar cross section, significant length variations as a function of orientational rotation angle will be observed if the bar is not perfectly straight. For the purposes of this discussion and all subsequent derivations, a ball bar artifact will be modeled as a simply supported beam with circular cross section. (See Fig. 2.) Ignoring the small changes in area of the cross section where the sphere is attached to the bar has a negligible effect on the model predictions. In the derivation of this model the terms *gauge point* and *sphere center* are used interchangeably as their functions as measurement reference points are analogous as described above.

**Fig. 4:**
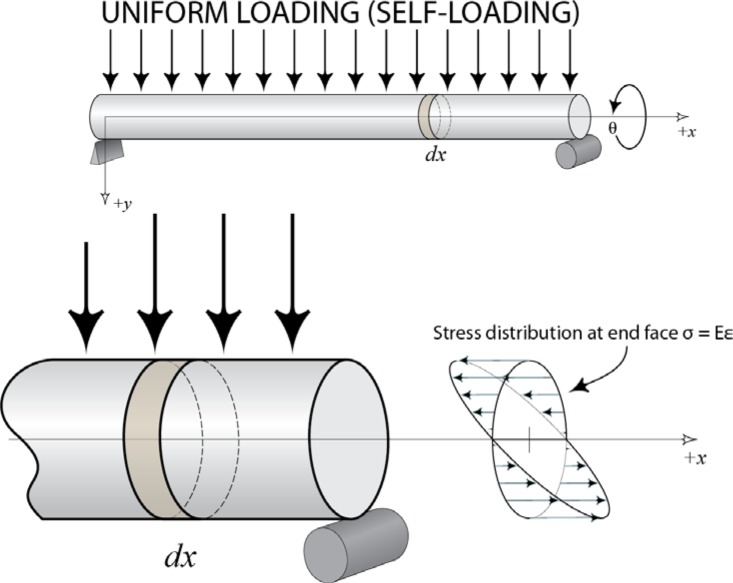
Distribution of the stresses at one end face due to internal bending moments.

If a straight beam is supported at any two points, internal stresses in the beam develop due to self (gravity)-loading. Specifically, when a beam is supported near its ends, the top surface of the beam experiences compressive stresses and the bottom surface tensile ones. This is shown in the depiction of the stress distribution at the end face of the beam shown in Fig. 4 [1]. At a specific point in each cross-section, the axial stress is zero. The collection of these points is frequently referred to as the neutral surface. If a straight ball bar is measured while both spheres are positioned above the neutral surface, the ball bar will measure physically shorter than it would if both spheres were positioned below the neutral surface — lengths *L*_1_ and *L*_2_ on the right side of Fig. 2. For a beam in pure bending with an external axial load, this behavior is modeled using equations from mechanics of materials, which describes the relationship between the bending moment, axial loads and the total stress in a beam [4, 8].

(1) $$\sigma_{\text{total}}=\frac{My}{I}+\sigma_{\text{due to axial load}}$$

Here, σ_total_ is the engineering stress at each end of the bar in the *x* direction; *M* is the internal bending moment in the bar; *y* is the vertical distance from the centroid of the cross section of the beam to the line joining the sphere centers or gauge points; *I* is the area moment of inertia of the ball bar cross-section; and σ_due to axial load_ is the stress due to external and gravitational loading along the axial or *x*-direction in Fig. 2. The first term on the right side of Eq. (1) describes the stress due to the bending moment and is obtained directly from beam tables and is widely accepted as a reasonable estimate of the stress distribution inside a beam in the presence of a uniform bending. Because there are no external forces in the *x*-direction and the beam is measured in the horizontal orientation, σ_due to axial load_ = 0. Consequently, the point along each face at which the total stress in the beam equals zero corresponds to *y* = 0. This implies that the neutral surface passes through the centroid of the cross-section.

Because the model is a simple case of uni-axial stress, the following equation can be used to describe the relationship between engineering stress, *σ*, and strain, *ε*. (Hooke’s Law for elastic materials)

(2) $$\varepsilon =\frac{\sigma_{\text{total}}}{E}=\frac{My}{IE},$$

where, *E* is the modulus of elasticity. Given that the engineering strain, *ε*, in the *x*-direction is simply the change in length divided by the original unstressed length, *∆L/L*, Eq. (2) can be written as follows:

(3) $$\Delta L=\frac{MyL}{IE}.$$

This equation is valid for the case of pure bending, i.e., when the internal bending moment is constant throughout the beam. For a perfectly straight beam under self-loading the internal bending moment is a function of the distance, *x*, along its length; the cross-sectional area moment of inertia; and the position of the supports. However, because the bending moment is continuous and slowly varying, it can be considered constant over the extent of each differential element of the beam. Using this observation and the fact that *y* is constant for a straight beam, Eq. (3) can be modified as follows:

(4) $$\Delta L=\frac{y}{IE}\int_{0}^{L}M\left(x\right)dx.$$

So, for the case of a straight beam with its gauge point offset from the neutral surface, *∆L* can be determined by measuring the vertical offset from the neutral surface and calculating the bending moment due to gravity. For a ball bar, the measurement of the offset *y* can be performed quite easily using two kinematic nests and a dial indicator as described in the *Measurement Procedures* section later in this paper.

Equation (4) implies that a straight ball bar supported at other than the Airy points should have the gauging spheres positioned precisely along the centroid of the bar axis, *y* = 0. This effect is largest for long ball bars that are supported on the gauging spheres which are near the ends of the bar and far from the Airy points. Specifically, a 1 m ball bar made of stainless steel with a 16 mm diameter cross-section supported as described may change in length by as much as 4 μm if both spheres are offset 1 mm (in the same direction) from the neutral surface. While it may seem unrealistic to encounter such a large geometric error in a commercially available ball bar—this is not always the case. For example, some ball bars have a flat machined along the full length of the ball bar to make it easier to attach a flat disc-shaped temperature sensor directly to the ball bar during measurement. Because gauging spheres are placed along the centroid of the tapered circular cross-section at the end of the ball bar, the flat effectively moves the centroid of the ball bar more than a full millimeter from the measurement line between the two spheres. Consequently, a straight 1 m ball bar designed as described above will change in length by as much as 4 μm due to this effect alone.

**Fig. 5:**
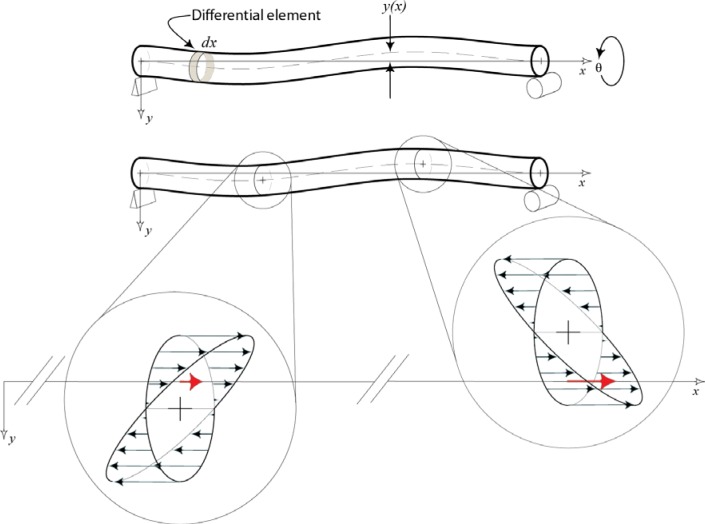
This illustration depicts the geometry of a ball bar model with curvature. The curvature is exaggerated for clarity. Also shown is the stress distribution at two cross-sections. Highlighted in red is the strain at each cross section that contributes to the variation in length of a ball bar.

Now consider the case where a ball bar is curved. The vertical offset, *y*, from the measurement line constructed through the gauging sphere centers to the neutral surface is a function of the distance along the ball bar, *x*, and the angle about the x-axis, θ. (See Fig. 5). Modifying Eq. (3) and integrating over the length of the beam leads to Eq. (5).

(5) $$\Delta L=\frac{1}{IE}\int_{0}^{L}M\left(x\right)y\left(x,\theta \right)dx.$$

Equation (5) is the expression for change in length of the ball bar as a function of vertical offset from the centroid of each cross-section to the measurement line joining the two spheres. This expression is more general than Eq. (4) and will be used for the remainder of this analysis. Substituting *y*(*x*, *θ*) = *y_k_* + *y*_1_(*x*, *θ*) into Eq. (5) and factoring leads to the following equation. Here, *y_k_* is a constant and *y_1_*(*x, θ*) are the values of *y*(*x, θ*) that are explicitly dependent on *x* and the angular orientation, θ.

(6) $$\Delta L=y_{k}\frac{1}{IE}\int_{0}^{L}M\left(x\right)dx+\frac{1}{IE}\int_{0}^{L}M\left(x\right)y_{1}\left(x,\theta \right)dx$$

The first term on the right side of Eq. (6) is identical to Eq. (4). This term describes the beam length change discussed above. The second term describes the change in length due to curvature in a ball bar, i.e., a non-straight bar. It is important to note that Eq. (6) implies that even if the spheres are centered on the end faces of the ball bar, in the presence of curvature, length variations may still be present. In fact, all the ball bars we observed that motivated this paper had the gauging spheres sufficiently centered on the end of the ball bars so that the first term on the right hand side of Eq. (6) was negligible. Hence the second term was the predominant source of the length change error observed in the ball bars.

An expression for the internal bending moment in the beam is required to utilize Eq. (6). These internal moments are a function of both the locations of the supports and the total loading on the beam. Again, the method in which a ball bar is supported during a measurement has a direct impact on the relationship between the length variation and angular orientation. The prescribed method for holding a ball bar during measurement is to place the support spheres into two kinematic nests: one, is a three-ball nest that constrains three translational degrees of freedom and the other a two-cylinder nest that constrains two rotational degrees of freedom. Because of the wide array of ball bar fixtures, a variable which describes the support location is included in the derivation of the internal bending moment in a ball bar. This allows the model to accommodate varied support positions.

**Fig. 6:**
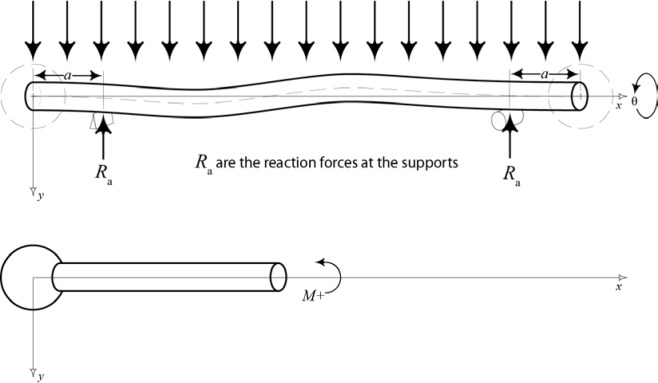
This figure shows the kinematic ball bar model employed as well as the sign convention for internal bending moments *M*_1_(*x*), *M*_2_(*x*), and *M*_3_(*x*) in Eqs. (7a–7c).

Figure 6 provides the force diagram that is used for the derivation of this ball bar bending model. The Variable *a* is the distance from the end of the beam to the closest support point; *R_a_* is the reaction force at each support and is equal to ½ the weight of the ball bar (assuming symmetrically placed supports and a bar that is not grossly out-of-straight); and *q* is the weight per unit length of the bar. Using the definitions above, the expressions for the internal bending moment of our ball bar model were derived, the results of which are provided below.

(7a) $$M_{1}\left(x\right)=-\frac{qx^{2}}{2}\;\text{for}\;0<x\leq a$$

(7b) $$M_{2}\left(x\right)=\frac{qL\left(x-a\right)}{2}-\frac{qx^{2}}{2}\;\text{for}\;a<x\leq L-a$$

(7c) $$M_{3}\left(x\right)=-\frac{qL^{2}}{2}-\frac{qx^{2}}{2}+qLx\;\text{for}\;L-a<x\leq L$$

The weight of the individual support spheres (Fig. 1) has been omitted. This is reasonable because the spheres are sufficiently close to the supports that they contribute very little to the internal bending moment in the ball bar and their weight is partially accounted for by taking *L* as the center-to-center distance. More specifically, a portion of the sphere is modeled as part of the bar material; comparison to experimental measurements show this assumption is valid. Combining Eqs. (7) and (6) leads to the general expression, Eq. (8), that describes the relationship between the angular orientation and the length variation for a ball bar with slowly varying curvature supported as described above. In this context, slowly varying curvature implies changes in the cross-sections are small enough to ensure that no large changes in the internal bending moment are present. Although it may be possible to estimate the bending moment at discontinuities, the uncertainty in these estimated values generally increases significantly as the number and size of the anomalies increase and may render the uncertainty in the length change estimates sufficiently large as to be of no practical use. Most commercially available ball bars meet the slowly varying geometry requirement.

(8) $$\Delta L=\frac{1}{IE}\left(\int_{0}^{a}M_{1}\left(x\right)y\left(x,\theta \right)dx+\int_{a}^{L-a}M_{2}\left(x\right)y\left(x,\theta \right)dx+\int_{L-a}^{L}M_{3}\left(x\right)y\left(x,\theta \right)dx\right)$$

Combining Eqs. (7) and (8) leads to Eq. (9):

(9) $$\Delta L\propto \frac{q}{IE}.$$

That is, the elongation is proportional to the linear density, *q*, and inversely proportional to the cross-section moment of inertia, *I*, and Young’s modulus, *E*.

If *y*(*x, θ*) is constant and different from zero, as is the case for a straight rod with the measurement points offset from the neutral axis in the vertical direction, then Eq. (8) can be used to calculate the position of the Airy Points, for two support points. Setting Eq. (8) equal to zero and solving for the roots of *a* gives $a=\left(\frac{1}{2}\pm \frac{\sqrt{3}}{6}\right)L$. This is equivalent to a distance between the supports of $\frac{1}{\sqrt{3}}L$, which is the accepted value for a set of Airy Points as given in [3]. It is important to note that the curvature and orientation of the ball bar alone give no indication of whether a ball bar will lengthen, shorten or remain unchanged when rotated about its long axis. It is the combination of the ball bar curvature and the relative position of each sphere on the ball bar end-faces that determines this behavior. Hence a bent bar measured with the bend concave up, may appear to shorten, lengthen or not change at all depending on the positions of the gauging spheres and support points.

As described earlier, the actual ball bars in this study contained some curvature. Furthermore, all of the ball bars in this study had the spheres positioned close to the centroid of the cross-section of the bar at the end face. That is, the measured ball bar length changes described in the next few sections of this document are described solely by the curvature term of Eq. (6).

## 4. Measurement Procedures

In order to compare the model predictions with physical measurements, it is necessary to measure the variation in sphere-center-to-sphere-center length as a function of the change in rotation angle as well as the vertical offset of the centroid of each cross-section relative to the measurement line (the line between the gauge points or sphere centers). The experimental setup used to measure these quantities and the results of the measurements are presented in this section.

**Fig. 7:**
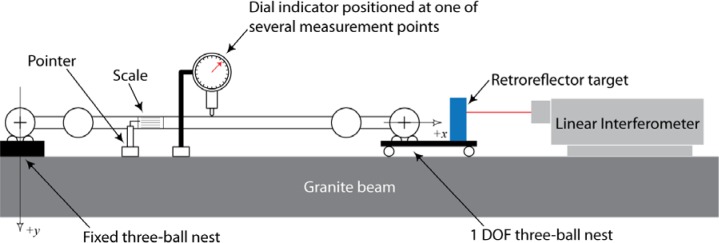
Experimental setup used to measure (1) the variation in center-to-center length for a specified change in angular orientation and (2) the offset of the centroid of each cross-section relative to the measurement line.

**Fig.8:**
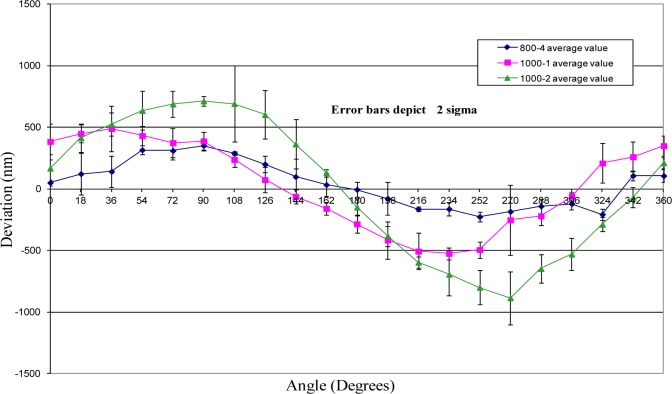
Shown are the length deviations from average value for 3 different ball bars.

**Fig. 9:**
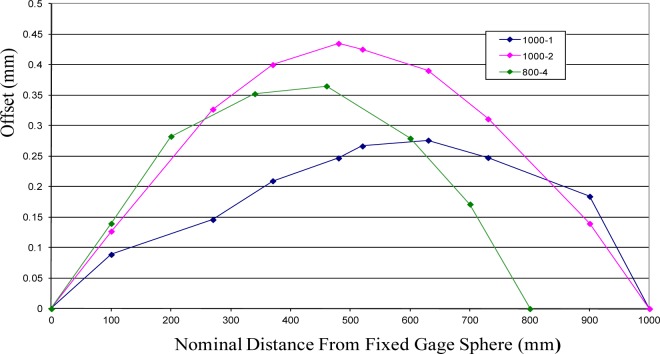
Shown are the values of the offsets of the centroid of sample cross sections from the measurement line between the gage spheres for 3 different ball bars.

A linear interferometer was used to measure the sphere-center-to-sphere-center length variation as a function of the rotation angle about the ball bar long axis. Each sphere of a ball bar was placed in one of two kinematic nests, which were composed of three small spheres. One nest was held fixed relative to the instrument table (granite beam), and the other was mounted on an air bearing carriage that can translate in the measurement direction. (See Fig. 7 and reference [9] for details.) A label with index marks at 18° increments was attached to the ball bar. (18° increments were selected as a compromise between the desire to measure very fine increments for more information content and the necessity to reduce the time of measurement to avoid thermal drift in the ball bar length.) A pointer mechanism was attached to the granite beam to ensure reproducibility of the angular alignment of the bar during measurements. Measurements were performed by recording the interferometer reading when the zero index was aligned with the index pointer. The bar was then rotated to each subsequent index position and the interferometer values recorded. This process was continued until the zero index mark was repeated. The complete measurement process described above was then repeated four times and the average value was the estimate of the length change, which is plotted in Fig. 8. The error bars in the figure represent two times the standard deviation of the four measurements at each measurement point. This measurement process was repeated for two 1000 mm ball bars and one 800 mm bar. The two 1000 mm ball bars (labeled 1000-1 and 1000-2 in the figure) were selected because the geometric shape of these two ball bars was very different and because long ball bars have greater sensitivity to sphere-center-to-sphere-center length change. One of the ball bars (1000-2) had larger curvature, and the curvature was nearly symmetric with respect to the mid-point of the ball bar. The geometry of the other 1000 mm ball bar was not symmetric, and the magnitude of the curvature was significantly smaller than the other 1000 mm ball bar. (See Fig. 9.) The 800 mm ball bar (labeled 800-4 in the figure) was selected to illustrate the model sensitivity to differences in ball bar length. The results of the length deviation from the average of the values are shown in Fig. 8.

The value *y*(*x, θ*) in Eq. (8) is the distance from the line joining the two gauging sphere centers to the horizontal plane containing the centroid of the corresponding cross-section. This component can be calculated from measurements at discrete locations along the ball bar, by simply placing a dial indicator at fixed positions. The indicator was aligned so that it measured displacement in the vertical direction. At index position zero the indicator was set to zero. The bar was rotated 18° and the value on the indicator recorded. This process was repeated for a full 360° rotation about the ball bar measurement axis. The dial indicator was then moved to other positions along the bar length and the above process repeated. The value for *y*(*x, θ*) at each measured position was obtained by subtracting the average of all the indicator readings at each position from the corresponding individual readings at the same location.

The geometry of each ball bar is depicted in Fig. 9. This figure shows the measured vertical distance between the plane containing the centroid of a cross section and the measurement line between the gauging spheres that contains the maximum value of *y*(*x, θ*) for each of the three ball bars used in this study. The figure also shows the unique variation in geometry for each of the ball bars. Further, the figure shows that the values for *y*(*x, θ*) are small relative to the length of each ball bar. Because of the slowly varying nature of *y*(*x, θ*) over the entire length of each ball bar, a simple piecewise linear function is used to approximate the values of *y*(*x, θ*) for any value of *x*. It is important to note that the maximum value of *y*(*x, θ*) for the ball bars in this study corresponded to a distance of approximately 450 µm. That is, the bend of each ball bar is sub-millimeter, hence, the bend does not have to be clearly visible to have consequential effects.

## 5. Results

**Fig. 10:**
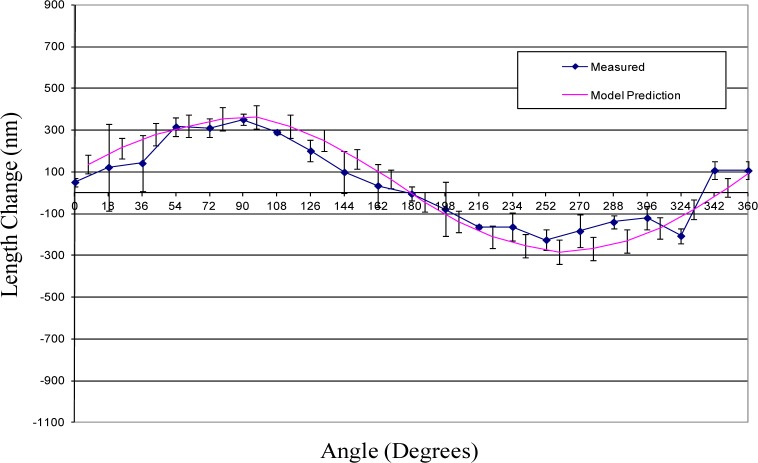
Shown is the length change from average value for ball bar serial number 800-4.

**Fig. 11:**
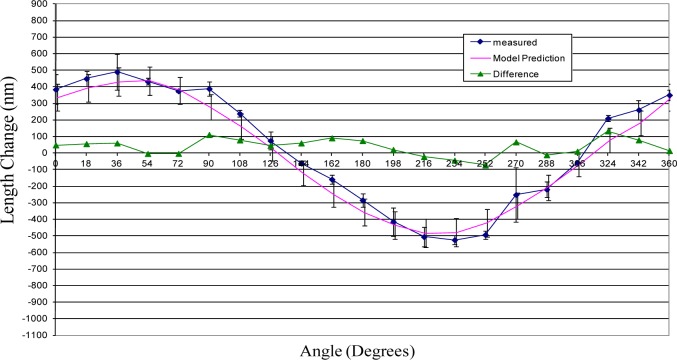
Shown is the length change from average value for ball bar serial number 1000-1.

**Fig. 12:**
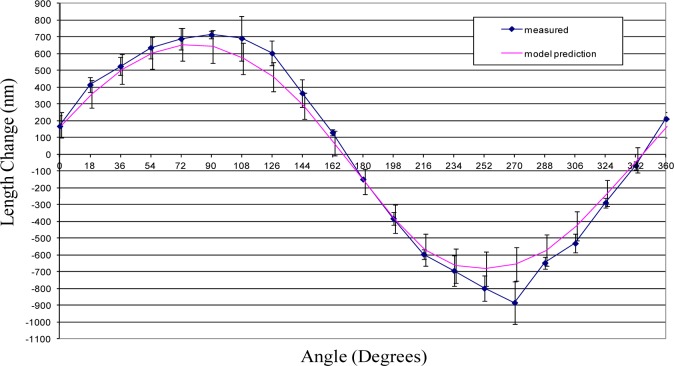
Shown is the length change from average value for ball bar serial number 1000-2.

This section provides the results of the comparison between the error model prediction and the actual measured sphere-center-to-sphere-center length variation for each of the three ball bars. The comparison between the measured and predicted length variations are depicted graphically in Figs. 10–12.

The uncertainties in the model predictions are represented by error bars in these figures. These error bars depict an expanded uncertainty, *U*, with *k = 2*. The relevant uncertainty components are provided in the Table 1. Each component is treated as uncorrelated with uniform distribution and the estimate for upper and lower bounds of the distribution and the corresponding standard uncertainties [10] are provided in the table as well. For most components the bounds are estimated from practical knowledge and through consultation with experts. The effects of the uncertainty in the support positions was sufficiently small that it is negligible and, consequently, not considered in this paper. Because the value for modulus of elasticity has a large effect on the model prediction, its value was determined experimentally.

The model predictions show good agreement with actual length variations. (See Figs. 10–12.) Initial predictions using a value for the modulus of elasticity obtained from a standard reference book underestimated the amount of length change. The experimentally determined value for modulus was approximately 10 % smaller than the average value obtained in materials handbooks.

There are also measured deviations that depart significantly from the theoretical predictions. The point in Fig. 12 at 270° is one example were the measured length change differs significantly from the theoretical model. Part of this difference may be due to sphere form error—departure from perfect spherical shape. That is, measurements on a straight 300 mm ball bar showed random variability in length of approximately 90 nm. This may also explain slight discrepancies between the measured and predicted results in Fig. 10 as well. The experimental setup employed three point kinematic nests to support the spheres during testing. The location of the sphere center can vary when using these nests if the sphere form error is sufficiently large. This variation in the sphere center locations can appear as an apparent change in length which is indistinguishable from geometry induced change.

Figures 10–12 show that in all cases the range of sphere-center-to-sphere-center length deviations after applying the model correction is less than 0.3 µm. The corresponding range for the uncorrected sphere-center-to-sphere-center lengths is approximately 1.5 µm. This implies that a large portion of the systematic length variation can be described by the model in this paper.

## 6. Conclusion

This paper shows that the measured length of long artifacts with imperfect geometry depends on the rotational orientation of the artifact, its mounting, and its construction. A systematic method to quantify these effects is also presented. The information presented here should be carefully considered when mounting artifacts for performance evaluation of CMMs and by metrologists during calibration of the artifacts for subsequent use. Designers of these artifacts should carefully consider implications of imperfect geometry and mounting of the artifact.

In some cases, heat treatment of these artifacts, which is performed to increase long term dimensional stability, can lead to slight bends in the bar as a result of asymmetric stress relaxation. In order to reduce length variations when a bend is present the linear density, *q*, to cross-section moment of inertia, *I*, should be minimized. For circular cross-sections, *I* is proportional to the radius to the fourth power and *q* is proportional to the square of the radius. As a consequence, the ratio of *q/I* will decrease as the radius increases. So a design engineer might consider specifying a larger diameter bar when constructing these artifacts. For hollow circular cross-sections *q* is proportional to the difference in the squares of the outside and inside radii while *I* is proportional to the difference in the fourth power of the radii. Therefore, artifacts constructed from tubes, as opposed to solid bars, have a smaller *q/I* ratio which also reduces the elongation due to self loading. Lastly, the ratio of q/E, where E is Young’s modulus, can be decreased by choosing a material with higher stiffness to weight ratio. However, other factors such as dimensional and environmental stability should be considered when choosing alternative materials to construct these artifacts.

## Figures and Tables

**Table 1:** Sphere-center-to-sphere-center error model influence quantities and estimated standard uncertainties

Input Quantity	Upper bound (*a*_+_)	Lower Bound (*a*_−_)	Standard Uncertainty (*u*(*x_i_*))
*y(x) (µm)*	25.4	−25.4	14.7
*I (mm^4^)*	40	−40	23
*E (N/m^2^)*	1×10^10^	−1×10^10^	5×10^9^
*A (mm^2^)*	0.12	−0.12	0.07
*q (N/m)*	3.8×10^3^	−3.8×10^3^	2.2×10^3^
